# Studying Autophagy in Zebrafish

**DOI:** 10.3390/cells6030021

**Published:** 2017-07-09

**Authors:** Benan John Mathai, Annemarie H. Meijer, Anne Simonsen

**Affiliations:** 1 Department of Molecular Medicine, Institute of Basic Medical Sciences, University of Oslo, Sognsvannsveien 9, 0317 Oslo, Norway; b.j.mathai@medisin.uio.no; 2 Institute of Biology Leiden, Leiden University, Einsteinweg 55, 2333 CC Leiden, The Netherlands; a.h.meijer@biology.leidenuniv.nl

**Keywords:** autophagy, zebrafish, GFP-Lc3, confocal microscopy, mitophagy, aggrephagy, xenophagy

## Abstract

Autophagy is an evolutionarily conserved catabolic process which allows lysosomal degradation of complex cytoplasmic components into basic biomolecules that are recycled for further cellular use. Autophagy is critical for cellular homeostasis and for degradation of misfolded proteins and damaged organelles as well as intracellular pathogens. The role of autophagy in protection against age-related diseases and a plethora of other diseases is now coming to light; assisted by several divergent eukaryotic model systems ranging from yeast to mice. We here give an overview of different methods used to analyse autophagy in zebrafish—a relatively new model for studying autophagy—and briefly discuss what has been done so far and possible future directions.

## 1. Introduction

**Figure 1:**
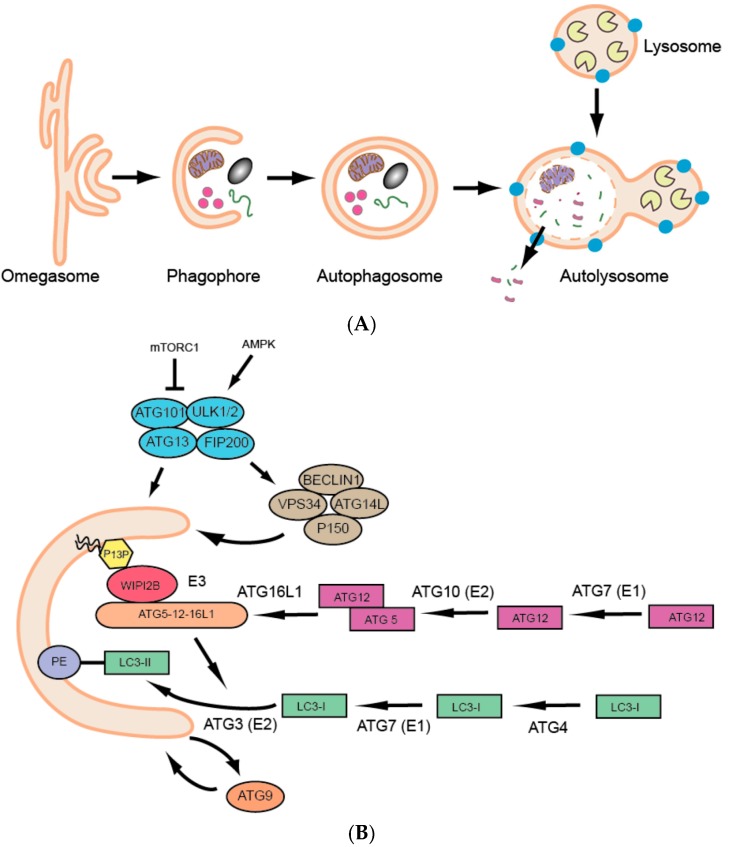
(**A**) Schematic overview of the process of macroautophagy; (**B**) Schematic overview of the core autophagic proteins involved in autophagosome biogenesis.

Over the past few decades we have seen a dramatic surge in research on a basic and fundamental cellular process called autophagy. Autophagy is defined as the lysosomal degradation of cytoplasmic materials (proteins, lipids, organelles, etc.), and three major types of autophagy have been described: macroautophagy, microautophagy and chaperone-mediated autophagy [1]. This review will focus on macroautophagy (hereafter referred to as autophagy), which involves the sequestration of cytoplasmic components in a double membranous structure, the autophagosome, followed by its fusion to the acidic lysosome, resulting in cargo degradation and release of simple biomolecules that can be reused for varied cellular purposes (Figure 1A). Thus, autophagy is an adaptive catabolic process leading to substrate formation for further anabolic energy-generating processes, to ultimately maintain homeostasis at the cell, tissue and organism levels. 

The molecular era of autophagy started with a series of genetic screens performed on unicellular yeast in the 1990s [2,3,4,5], which were followed by the identification of respective homologs in higher eukaryotes and resulted in the characterization of more than 30 AuTophaGy-related (ATG) genes [6]. The ATG proteins essential for autophagosome formation are referred to as the ‘core’ autophagy machinery (Figure 1B) [7] and include: (1) the UNC-51-like kinase (ULK) complex composed of ULK1 or ULK2, ATG13, ATG101 and FIP200; (2) the class III phosphatidylinositol 3-kinase (PI3K) complex (PIK3C3), consisting of the catalytic subunit VPS34, as well as BECLIN1, p150 and ATG14L; (3) the two ubiquitin-like conjugation systems that lead to the conjugation of ATG12 to ATG5 and ATG8 to phosphatidylethanolamine (PE) in the phagophore membrane and finally (4) the transmembrane protein ATG9 [8,9]. Human ATG protein names are used here, see Table 1 for the respective zebrafish ATG orthologue names. 

In addition to these core autophagy proteins, the regulation and execution of the pathway is tightly controlled by a large number of proteins and lipids and we are only beginning to understand how their interconnections are regulated in time and space under various metabolic conditions and in different tissues. Dysfunctional autophagy is closely associated with tumorigenesis [10,11], immune disorders [12], neurodegeneration and aging [13], infectious diseases [14] and diabetes [15]. Thus, a detailed understanding of the molecular mechanisms involved in autophagy may open doors to various therapeutic approaches against diseases where autophagy plays an indispensable role. 

Our understanding of how autophagy is regulated under different physiological and pathological conditions is largely based on research performed in different tractable animal model systems such as the fruit fly *Drosophila melanogaster* [16,17], nematode *Caenorhabditis elegans* [18], the mouse *Mus musculus* [19,20], oysters [21] and *Dictyostelium discoideum* [22]. Recently, there has been an exponential interest in using zebrafish (*Danio rerio*) for varied research owing to the immense advantages that it offers. The small size, high fecundity, external fertilization, transparent embryos, rapid development, and genetic tractability of zebrafish make it highly desirable for basic science and translational high throughput research [23]. 

We here review the current literature and the methods used to study autophagy in zebrafish, including DNA, RNA and protein-based methods. We also discuss different types of selective autophagy, with emphasis on mitophagy, xenophagy and aggrephagy and how these can be studied in zebrafish. Finally, we provide detailed information about different antibodies, chemical reagents and reporter lines that have been used to analyze autophagy in zebrafish and discuss how current methods could be improved to better understand autophagy in zebrafish. 

## 2. Zebrafish Autophagy Genes

The identification of the zebrafish as a genetically tractable organism in the 1980s led to its immense usage in the 1990s, whereby a large number of mutations giving rise to specific phenotypes were discovered through large-scale mutagenesis screens [24]. However, this alone was insufficient to throw light on various rare and common human disorders as a high-quality zebrafish genome sequence and complete annotation of zebrafish protein-coding genes with identification of their human orthologues was limited. The genome of the zebrafish has now been published as a well-annotated reference genome, providing key insights into the use of this vertebrate as a desirable model to mimic human disease states. In total, 84% of human disease-associated genes have at least one obvious zebrafish orthologue [25,26,27].

To be able to alter or modulate autophagy genetically in zebrafish, it is critical to delineate the representative ATG zebrafish orthologues from its yeast or mammalian counterparts. We searched for human ATG proteins from National Centre for Biotechnology Information (NCBI) and blasted their respective amino acid sequences against *Danio rerio*’s (taxid: 7955) reference proteins as a search set. The hit with highest query coverage and smallest E-value was selected to be an orthologue. We also compared the sequence with that annotated in the Ensemble genome browser. A detailed account of the core ATG proteins (mammalian) and their respective zebrafish orthologue with Refseq IDs and Ensemble IDs have been tabulated (Table 1). The overall amino acid identity between human and zebrafish core autophagy proteins range between 40 and 96% (Table 1).

## 3. Genome Editing Techniques

Genome editing, or the idea of introducing a desired change to the genomic DNA sequence, is currently driving a revolution in the medical field with the introduction of the Clustered Regularly Interspaced Short Palindromic Repeats (CRISPR)/CRISPR Associated Protein 9 (Cas9) technology [28,29,30]. An ideally desirable genome editing tool would edit any genomic locus with high efficiency, specificity and with little or no off-target effects. The basic process of nuclease-based genome editing is to create a specific double-strand break (DSB) in the genome and then allow the cell’s own endogenous repair machinery to repair the break, by either non-homologous end-joining (NHEJ) or by homology-directed repair (HDR). The different techniques of genome editing used in zebrafish (CRISPR/Cas9, transcription activator-like effector nucleases (TALENs) and zinc finger nucleases) have been extensively reviewed elsewhere [31,32,33,34,35,36,37]. We will here discuss how genome editing could help drive the field of zebrafish autophagy.

### 3.1. CRISPR/Cas9 Mutagenesis 

The CRISPR/Cas9 technology has been widely adopted in the zebrafish community and has already come a long way from the first knock-out [38], to high-throughput mutagenesis screens [33], conditional knockout [39], multiplex knockout [40,41] and to targeted insertion of DNA elements [42]. It would be highly desirable to apply systematically all of these techniques into understanding the precise role of autophagy proteins in zebrafish development, physiology and pathology. 

Briefly, CRISPR/Cas9-mediated genome editing in zebrafish is facilitated by the microinjection of a “short guide-RNA” (sgRNA) and Cas9 endonuclease protein into zebrafish embryo (at 1 cell stage), wherein the Cas endonuclease protein, forms a complex with the sgRNA molecule (now called the Cas9 holoendonuclease). Cas9 holoendonuclease or the corresponding RNAs (sgRNA + Cas9 messenger RNA (mRNA)) can be injected. The target DNA sequence, in addition to being complementary to the gRNA molecule, should also have a “protospacer-adjacent motif” (PAM), that is required for compatibility with the particular Cas protein being used. Once mobilized to the target DNA site, the Cas9 holoendonuclease generates a double-strand break (DSB), which can be used to create a knock-out or add a specific function to a gene (targeted knock-in). Autophagy can be manipulated by injecting sgRNA against the core autophagy genes (Table 1) together with either Cas9 mRNA or protein. It is very important to minimize or best, to negate, mutagenesis of an incorrect gene (off-target effect). Step-by-step protocols describing how to design an efficient sgRNA and the heuristic rules surrounding it, purifying Cas9 mRNA or using commercial Cas9 protein along with sgRNA have been reviewed previously [43,44]. The transparency of zebrafish larvae makes zebrafish highly desirable to use for generation of reporter lines. CRISPR/Cas9 can be used to tag core autophagy genes endogenously by “knocking-in” a reporter DNA element upstream/downstream of the autophagy gene of interest, e.g., to generate a fusion protein at an endogenous locus. This is highly desirable in the study of autophagy, opening up the prospect of “double-tagging” an autophagy protein or a cargo of interest and following their degradation kinetics. “Double-tagging” is based on the principle of using tandem fluorescent tags, where one will be quenched (e.g., green fluorescent protein (GFP)) upon delivery to the acidic lysosome. CRISPR/Cas9-mediated genome editing can also used to ablate a particular gene in a specific tissue or at a particular developmental time-point. As an example, *LoxP* sites can be “knocked-in” to flank an autophagy gene of interest and later by using the cre recombinase, the gene can be inverted or excised, thereby creating a complete knock-out. This is suitable for genes whose knockout can be embryonically lethal. 

The use of CRISPR/Cas9-based targeted mutagenesis for deriving stable transgenic zebrafish or zebrafish knockout autophagy lines is in its initial phase. So far only one study has used this system to create mutant lines. CRISPR/Cas9-based mutagenesis in *spns1* and *atp6v0ca* genes induced premature autophagosome-lysosome fusion marked by insufficient acidity leading to developmental senescence and death [45]. *spns1* is thought to function as a lysosomal H^+^-carbohydrate symporter, which functions at a late and terminal stage of autophagy [46,47]*. atp6v0ca* encodes a sub-unit of the vacuolar-type H^+^-ATPase (v-ATPase) that counteracts *spns1* ablation effects in zebrafish. It is highly likely that we will soon see increasing use of CRISPR/Cas9 technology to modulate autophagy in zebrafish. 

### 3.2. TALENS and ZFNs

Since the introduction of CRISPR/Cas9 for genome editing in zebrafish, the use of TALENs and ZFNs, which were used before for genome editing [36,37] have taken a back seat (for a review of these methods see references). The use of TALENs and ZFNs to study autophagy in zebrafish is limited. TALEN-mediated mutation of the nuclear hormone receptor *nr1d1* was shown to have a positive effect on autophagosome-autolysosome number and lead to upregulation of ATG genes. *nr1d1* mutants were also shown to affect the circadian clock by significantly upregulating the circadian clock genes, leading to the conclusion that the circadian clock regulates autophagy rhythms in zebrafish larvae [48].

### 3.3. Transient Gene Knockdown by Morpholino Oligonucleotides

Morpholino oligonucleotides or morpholinos, first developed by Dr. James Summerton, are oligomers of 25 morpholine bases that are targeted via complementary base pairing to the mRNA of interest. They silence the gene by either blocking the translational start site from the ribosomal machinery or by blocking the splice sites (donor/acceptor), thereby interfering with the binding of spliceosome components [49,50]. Morpholinos can be used to interrogate pathways and associate genes with a phenotype and this can be done easily by just injecting an optimal volume of the morpholino solution into the yolk sac of a zebrafish embryo at the 1–4 cell stage. Morpholinos provide precise spatial targeting of multiple gene products [51] and are extremely useful for silencing and analyzing maternal gene expression [52]. However, a drawback of morpholinos is the relatively frequent off-target effects. Off-target effects are often caused by the induction of p53 that leads to apoptosis, but can also be p53-independent [53,54]. Inconsistencies between morphant and CRISPR mutant phenotypes have been seen in some studies [54], whereas others have shown that such inconsistencies can be explained by a compensating gene that is upregulated in the mutants, but not in the morphants [55]. Recent reports point out off-target single nucleotide variations (SNVs) in CRISPR-repaired mice, fished out via whole genome sequencing (WGS) [56]. Therefore, if used with the appropriate controls, morpholinos remain a useful tool [57].

Morpholinos have been employed vigorously to analyze autophagy in zebrafish and have provided valuable insight into the role of autophagy in development and disease. Knockdown of Atg5, Atg7 and Beclin1 [58,59], Atg4da [60], Ambra1a and Ambra1b [61,62] all show an important role of autophagy during embryogenesis. One of the common phenotypes seen consistently among these studies is a cardiac defect, indicating a very specific role of autophagy in cardiac morphogenesis/function, in alignment with previous studies on rodents [63]. Moreover, knockdown of optineurin, an ubiquitin-binding autophagy-receptor protein, was shown to cause motor axonopathy due to defective autophagic clearance of accumulated SOD1-G93A aggregates [64], defective vesicle trafficking in the axons [65], and increased susceptibility to *Salmonella enterica* infection [66]. Morpholino-mediated depletion of Spns1, a lysosomal transporter, was found to upregulate embryonic cellular senescence [46] and this was counteracted by the depletion of the lysosomal v-ATPase, which together suppresses developmental senescence and increases life-span [45]. Transient depletion of p62/sqstm1, another ubiquitin-binding autophagy receptor protein, in zebrafish embryos was shown to increase susceptibility to *Shigella flexneri* and *Mycobacterium marinum* in the host, indicating the role of autophagy against bacterial infection [67,68]. In another study involving the knockdown of p62/sqstm1 in zebrafish, it was seen that the ablation caused a specific locomoter phenotype characterized by a specific axonopathy of descending motor neuron projections [69]. Sorting nexin 14 knockdown in zebrafish larvae led to neuronal cell death (neurodegeneration) associated with defective autophagic degradation, ultimately resulting in cerebellar ataxias [70]. 

Several reports have indicated an indirect escalation or enervation of autophagy in zebrafish models of gene ablation by morpholinos. Zebrafish embryos depleted of the phosphatidylinositol 3-phosphatase *MTMR14 (*better known as Jumpy) showed an increase in autophagy at 1 day post fertilization (dpf) [71], consistent with previous results in mammalian cells, showing that MTMR14 dephosphorylates PI(3)P in the early autophagic membranes, thereby inhibiting autophagy [72]. We recently found that the PX domain protein *Hs1bp3* also regulates the formation of autophagosomes by a novel negative-feedback mechanism on membrane lipids. Morpholino-mediated depletion of *Hs1bp3* in zebrafish embryos caused an increase in GFP-Lc3 puncta, which was rescued by co-injection of mRNA encoding the human HS1BP3 protein, thereby validating the conserved role of *Hs1bp3* as negative regulator of autophagy in vivo [73] (Figure 3). 

In another study, depletion of collagen VI (*COLVI*), a protein crucial for structural integrity, cellular adhesion, migration and survival, resulted in reduced lipidation of Lc3 and reduced expression of Beclin1, suggesting an overall inhibition of autophagy in these morphants, ultimately leading to muscle dysfunction [74]. The role of autophagy in survival of hematopoietic cells was observed in a disrupted ribosome biogenesis model of zebrafish where *rps19* was ablated using translation morpholino [75,76]. A detailed list of all morpholinos used to analyze autophagy in zebrafish has been included in another review [77].

### 3.4. Mutations

In a major effort to generate mutant zebrafish lines, Christiane Nüsslein-Volhard and Wolfgang Driever orchestrated two of the largest mutagenesis screens ever performed in zebrafish [24,78]. These studies brought forth about 1500 mutations in more than 400 genes, but neither these original screens nor any later screens have revealed a mutant allele of a core autophagy gene. One possible reason for this could be that such mutations would be early embryonic lethal or it might be explained by the late onset of autophagy-related phenotypes in zebrafish.

A high quality sequence assembly of the zebrafish genome was initiated by the Sanger Institute (UK) in 2001 and completed in 2013 [25]. The Sanger Institute also initiated a systematic effort called the Zebrafish Mutation Project (ZMP) [26], which has created mutant alleles in over 16,000 protein-coding genes, including a number of core autophagy genes (Table 1). Using such autophagy mutant lines would provide valuable insight into the role of autophagy in physiological processes.

## 4. RNA-Based Analysis

Autophagy is known to be tightly regulated by posttranslational modifications of autophagy proteins (e.g., phosphorylation of ULK1 by mTORC1 and AMPK oppositely regulate the activity of the ULK1 complex) and by regulation of protein levels. But in order to obtain a real estimation of autophagy it is necessary to also monitor their mRNA levels [79]. It is however important to note that increased mRNA levels of autophagic genes should not be interpreted as increased autophagy, as it can be a compensatory mechanism. A detailed list of primers used to assess the expression of autophagy-related genes by quantitative real-time PCR (qRT-PCR) in zebrafish has been reviewed recently [77]. Zebrafish embryos and larvae are also very suited for whole mount in situ hybridization (WISH), which provides information about the spatial expression of a particular gene in the whole organism. This does not aid much in answering questions on autophagy activity, but still could help analyze the spatial arrangement of autophagy genes under certain conditions. WISH expression patterns are systematically catalogued in the zebrafish information network (ZFIN) database (zfin.org). 

**Figure 2:**
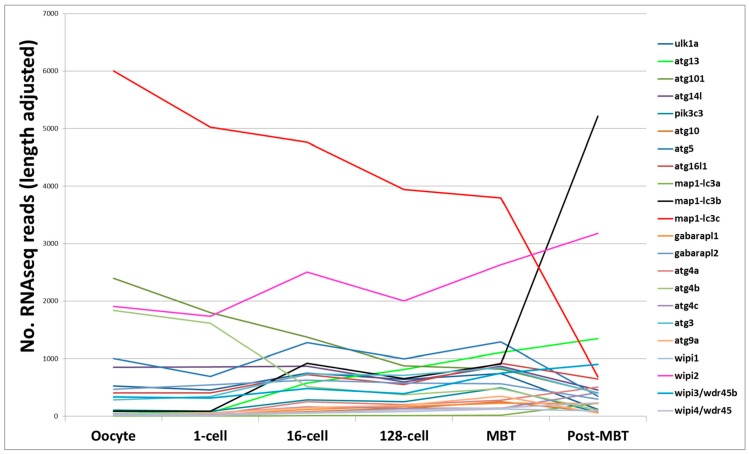
Messenger RNA sequence (mRNA-seq) analysis. Line plot of core autophagy gene transcripts analyzed by mRNA-seq in zebrafish embryos from the oocyte stage to post-mid blastula stage transition.

mRNA sequencing is a sensitive and accurate method for analyzing the transcriptomes of disease states and/or of biological processes. Prior to the activation of the zebrafish embryo genome, maternally-derived mRNA regulate early development in zebrafish [80,81]. This occurs at the 10th cell division (~3.5 h post-fertilization) when the zebrafish zygotic genome gets activated, also known as the mid-blastula transition (MBT) [82]. Mathavan and colleagues applied mRNA deep sequencing (mRNA-seq) to gain a comprehensive understanding of all transcriptional processes occurring from the unfertilized egg to early gastrulation [83]. We procured the raw data and fished for “core autophagy genes” in the data (available in the Gene Expression Omnibus (GEO) database, accession number GSE22830). Almost all of the core autophagy genes are expressed maternally at quite low levels, except for *map1-lc3c* which is expressed at high level from the oocyte to MBT. Interestingly, while the expression of *map1-lc3c* tapers off post MBT, there is a correspondingly strong increase in *map1-lc3b* expression levels at MBT, suggesting that *map1-lc3c* plays an important role during the early embryonic cell divisions, with *map1-lc3b* being more important later. *Wipi2* is consistently highly expressed across the early cell divisions to gastrulation (Figure 2). Several other mRNA-seq datasets are publicly available in the GEO database, also covering other later development stages. For example, in a developmental time series from 1 to 6 days post fertilization it was shown that the autophagy modulator gene *dram1* is upregulated during *Mycobacterium marinum* infection [84]. 

## 5. Protein-based analysis

### 5.1. Fluorescence Microscopy 

**Figure 3:**
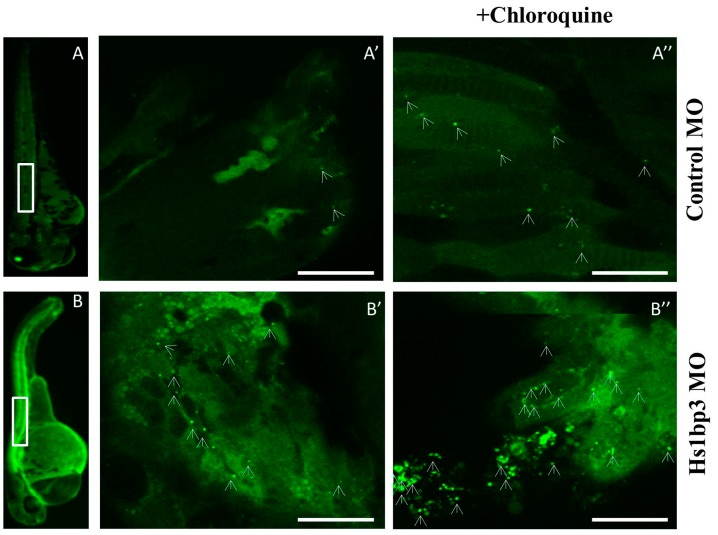
Confocal imaging of Tg(CMV:GFP-Lc3). Representative confocal images of GFP-Lc3 puncta (autophagosomes) in the trunk area of GFP-Lc3 transgenic zebrafish embryos injected with control morpholino or Hslbp3 translational-blocking morpholino and imaged at 2 days post fertilization (dpf) with or without pre-treatment with chloroquine (10 mM) for 6 h. Scale bars, 10 µM for the confocal images. Panel A, B shows the whole zebrafish larvae at 2 days post fertilization highlighting the trunk area chosen for confocal imaging; Panel A’, A’’, B’, B’’ shows respective confocal images.

The most widely used marker to study autophagy is Atg8/Lc3, as this protein becomes conjugated to PE in the autophagic membrane upon induction of autophagy and remains bound throughout the pathway [85]. The lipidated form of Lc3 (called Lc3-II) can be visualized as cytoplasmic puncta by immunofluorescence microscopy or by a shift in molecular weight when analyzed by sodium dodecyl sulfate-polyacrylamide gel electrophoresis (SDS-PAGE) (described below). To analyze Lc3 lipidation in vivo, it is common to measure the increase in Lc3 puncta, using models where the N-terminus of Lc3 is tagged to a fluorescent reporter protein such as GFP. In zebrafish, GFP-Lc3 can be visualized in vivo during development due to the transparency of zebrafish embryos (Figure 3). Transgenic GFP-Lc3 and GFP-Gabarap fish have been generated [86] and are described in more detail below. Zebrafish larvae beyond 2 dpf develop pigments, which would be a hindrance for normal fluorescent microscopy. However, larvae can still be visualized for cellular activities in transgenic reporter lines by supplementing the media with 1-phenyl-2-thiourea (PTU), which inhibits melanogenesis or by using zebrafish strains that have mutations affecting pigment production [87]. Fusion of autophagosomes with lysosomes can be readily detected in vivo by the addition of LysoTracker Red to fish media prior to visualization [86].

Fluorescence methods (reporter lines or immunofluorescence (IF)) are more sensitive and quantitative as compared to molecular techniques like western blotting. Increased autophagic activity is usually marked by a significant change in the number of fluorescent puncta. However, it is very important to note here that an increase in GFP-Lc3 puncta can be caused by an increased flux or by impairment of autolysosome formation [79]. Therefore, proper flux experiments must be done (e.g., stimuli in the absence or presence of lysosomal inhibitors) to be able to conclude. While quantifying live in vivo images from a reporter line like the GFP-Lc3 line, it has to be done on a ‘per-cell area’ basis rather than simply using the total number (or percentage) of cells displaying puncta. This point is important as in zebrafish larvae which have a constant supply of nutrients from the yolk-sac, there could be some cells displaying a basal level of GFP-Lc3 puncta under “fed” conditions. In situations where endogenous Lc3 could be stained with a specific antibody, it is important to counterstain the nuclei with DAPI or Hoechst and then quantify puncta on a ‘per-cell’ basis. Another important caveat to be noted when using GFP-Lc3 is its tendency to bind to aggregates, especially when working with protein-aggregation models or when overexpressed [88]. Interpretation of autophagy in these models should be done by negating off the background fluorescence by having an untagged internal GFP control [79] or by the use of a C-terminal glycine mutant GFP-LC3 that is defective in ubiquitin-like conjugation with phosphatidylethanolamine, (GFP-LC3^G120A^ as a negative control) [89] or by another fluorescent protein tandemly fused to GFP, e.g., red fluorescent protein (RFP) (GFP-RFP-Lc3). Using lysosomal dyes (e.g., LysoTracker Red) in tandem with GFP-Lc3 is another useful approach [46,86,90]. Here the colocalization of GFP-Lc3 and LysoTracker can be used as an indicator of autophagy. 

The use of transgenic zebrafish models to study autophagy was kick-started by Klionsky and co-workers who developed the Tg(CMV:GFP-Lc3) and Tg(CMV:GFP-Gabarap) transgenic lines [86]. The Tg(CMV:GFP-Lc3) line has been used in various studies giving important insights into the functional significance of autophagy and autophagy modulators in vivo [46,58,67,68,73,91,92,93,94]. Tg(fabp10:EGFP-Lc3) and Tg(TαCP:GFP-Lc3) were used recently for looking into autophagy in the liver [95] and in photoreceptors [90], respectively. 

The introduction of the tandem fluorescent tagged Lc3 in mammalian cell lines [96] opened up for the possibility of making similar tandem tagged (e.g., RFP/mCherry-GFP) reporter lines in model organisms. As briefly mentioned above, the underlying principle of using a tandem tag to study autophagy is based on the pH sensitivity of GFP, with the GFP signal being quenched when the tagged protein reaches the acidic environment of the lysosome, while the red signal (RFP/mCherry) is stable. Thus, the ratio between yellow (autophagosomes) and red (autolysosomes) signal can readily be used to quantify autophagic flux. A recent study used a transgenic zebrafish line expressing the tandem tag for Lc3 under the control of a photoreceptor promoter, Tg(TαCP:mCherry-GFP-map1lc3b) [97]. The tandem-tag principle can be exploited to generate other zebrafish reporter lines (by tandem-tagging selective autophagy cargo or autophagic receptors) that would contribute to our understanding of autophagy and the mechanisms underlying its role in zebrafish development and physiology. 

As mentioned earlier, one should be cautious when interpreting Lc3 data. An increase in Lc3 levels should be validated by estimating the total autophagic flux, by e.g., treating samples with and without lysosomal inhibitors, such as Bafilomycin A1 or chloroquine (Figure 3). There are however reports that lysosomal inhibitors could inhibit mTORC1 and induce “unwanted” autophagy [98,99,100]. Taking these loopholes into consideration, the Mizushima group recently constructed a novel probe, GFP-Lc3-RFP-Lc3ΔG, which they tested in zebrafish as well [101]. The transgenic zebrafish line that ubiquitously expresses Tg(GFP-Lc3-RFP-Lc3ΔG) aided robust assessment of autophagic flux by the measurement of the GFP/RFP ratio. The underlying principle here is that the reporter probe will be cleaved by endogenous Atg4 proteases into equimolar amounts of GFP-Lc3 and RFP-Lc3ΔG, a mutant unable to become conjugated to the autophagy membrane. Thus, while GFP-Lc3 becomes lipidated and degraded by autophagy, the RFP-Lc3ΔG remains in the cytosol, serving as an internal control. Autophagic flux can then be estimated by calculating the GFP/RFP signal ratio. 

Fluorescent reporter lines of other autophagy core components or probes to detect autophagic membranes would also be desirable. The zebrafish transgenic reporter lines Tg(TαCP:YFP-2XFYVE) and Tg(TαCP:tRFP-t-2XFYVE) are examples of the latter. The FYVE domain is a conserved protein motif characterized by its ability to bind with high specificity to phosphatidylinositol 3-phosphate (PI(3)P), a phosphoinositide highly enriched in early endosomes, but also detected in early autophagic structures and found to be important for autophagy [102]. These zebrafish transgenic reporter lines (Tg(TαCP:YFP-2XFYVE) and Tg(TαCP:tRFP-t-2XFYVE)) were recently used to characterize endolysosomal trafficking events upon ablation of the polyphosphoinositide phosphatase, Synaptojanin1 *(synj1)* in cone photoreceptors [97]. A summary of the zebrafish autophagy reporter lines used in zebrafish can be found in Table 2. 

In cases where there are no stable reporter lines available and one wants to investigate autophagy during embryonic development (up to 5 dpf or depending upon the half-life of the transcribed mRNA), it is possible to inject in vitro transcribed mRNA for a reporter tagged to Lc3 or any other autophagy marker protein, such as mCherry-Lc3 mRNA in vitro transcribed from the vector pDest(CMV:RFP-GFP-Lc3) [104,105,106]. 

### 5.2. Western Blotting 

The most widely used method for analyzing autophagy is by measuring the levels of lipidated or membrane bound form of Atg8/Lc3B (Atg8-PE/Lc3B-II), as it runs at a different molecular weight than the cytosolic form of Lc3 (Lc3-I) by SDS-PAGE [79,107]. This method has been used to measure levels of autophagy in some zebrafish autophagy studies [46,58,86,95,104,106,108,109]. Again, it cannot be concluded that a mere increase in Lc3-II levels corresponds to increased autophagy, as this can also be due to autolysosomal formation defects, and it is therefore important to do proper autophagic flux experiments (as described above) to conclude about increased/reduced autophagy. 

It is very critical to differentiate between the lipidated Lc3-II and the unlipidated Lc3-I when immunoblotting for Lc3. As these two bands lie pretty close to each other (approximately 14 and 16 kDa), one can be masked by the other and this problem is intensified if the zebrafish embryo is not deyolked prior to preparing the lysate. The yolk sac is enriched with the protein Vitellogenin and this can cause overloading effects while blotting, if not removed by a deyolking buffer such as Ringer’s solution [110]. It is also critical to use gels that give a good separation in the 15 kDa area. 

Reproduction of Lc3 blots can be a major hindrance, primarily attributed to changes in experimental setups. The lysis buffer used, the incubation times for blocking, and the primary and secondary antibodies as well as washing periods should be optimized. The type of membrane used for blotting also makes a difference, as Lc3-II binds more effectively to the polyvinylidene fluoride (PVDF) membrane whereas nitrocellulose has a higher affinity for Lc3-I. It is also beneficial to dry the membrane for a short time after transfer to potentially stabilize the binding of Lc3 to the membranes. The following should also be taken into consideration while blotting for Lc3: sensitivity issues of Lc3-I to freeze-thawing (lysates should be run right after boiling), and comparison of Lc3-II levels to a housekeeping protein (e.g., actin or tubulin) rather than comparing them to Lc3-I*,* as Lc3-I levels can vary (e.g., upon cellular stress and from tissue to tissue) and not necessarily represent autophagy levels. Finally, it is necessary to also monitor the *lc3B* mRNA levels and to compare the correlation between protein Lc3B and mRNA *lc3B* [79].

Even though Lc3 remains the primary target to reveal levels of autophagy, other core autophagy proteins have also been studied. Knockdown of Atg5, Atg7 and Beclin1 in zebrafish were validated via Western blotting in a study aimed at investigating a possible role of autophagy during zebrafish embryogenesis [58]. Beclin1 levels were also examined in ambra-1 knockdown embryos [62]. A detailed list of autophagy related antibodies successfully used for Western blotting and immunofluorescence in zebrafish is shown in Table 3.

### 5.3. Transmission Electron Microscopy (TEM)

Autophagy was first discovered in the 1950s using transmission electron microscopy (TEM) [122]. TEM is a classical and widely used method to observe autophagic structures. If properly sampled, TEM provides superlative ultrastructural images with much higher resolution than any light microscope or super-resolution microscope. It gives details of cellular coats, cellular components and bodies in their natural environment [79,123].

TEM has been used to a limited extent in zebrafish autophagy research, owing to the difficulty in sampling and instrument availability. TEM has been used to demonstrate the presence of autophagosomes during zebrafish embryogenesis [58], during caudal fin [91] and muscle regeneration [94] and a variety of other contexts. For example, TEM revealed an increased number of autophagosomes and autolysosomes in the intestinal epithelial cells of zebrafish harboring a mutation in a ribosomal RNA processing gene, *pwp2h* [106]. Here increased autophagy enhanced survival of this zebrafish ribosomopathy model In contrast, aberrant autophagy was observed in a zebrafish motor dysfunction model [71], in Atrogin1-deficient zebrafish [117] and in a variety of zebrafish bacterial infection models [67,68,92]. The *Salmonella* plasmid virulence gene, *spvB*, was shown to enhance bacterial virulence by inhibiting autophagy [120]. 

## 6. Chemical/Pharmacological Modulations

**Figure 4:**
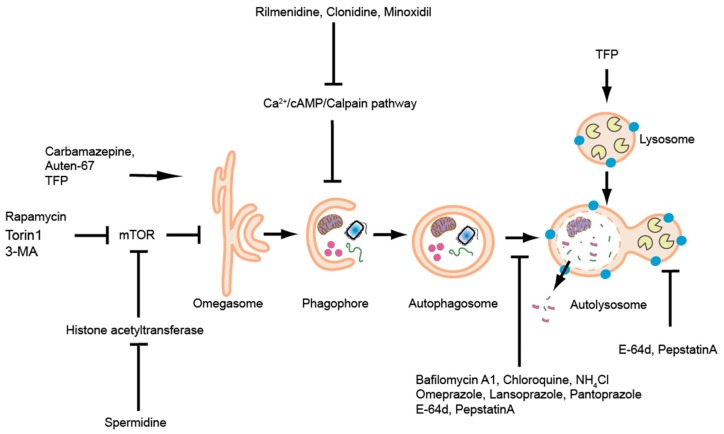
Schematic overview of the autophagic pathway and a partial list of reagents (reagents used beyond 2014, Table 2) that modulate autophagy in zebrafish are indicated.

Zebrafish embryos are easily treatable by waterborne exposure. Drugs that can modulate autophagic activity by either inducing it, decreasing it or blocking autophagosome-lysosome fusion have been well-used in zebrafish [124]. A detailed list of reagents used to interfere with autophagic activity in zebrafish (until 2014) has been reviewed previously [124]. We here present a list of autophagic modulators used in papers published after 2014 (Table 4, Figure 4).

## 7. Selective Autophagy

While induction of autophagy upon nutrient deprivation or other forms of stress is believed to be an unselective process when it comes to the types of cargo being sequestered and degraded to supply cells with essential building-blocks to survive the period of stress until cellular homeostasis is restored. Autophagy can however also be a highly selective process, with different cargo-specific sub-types, including lipophagy (autophagy of lipid droplets), ferrintinophagy (autophagy of iron bound ferritin), lysophagy (autophagy of lysosomes), reticulophagy (autophagy of ER), ribophagy (autophagy of ribosomes), xenophagy (autophagy of pathogens), aggrephagy (autophagy of protein aggregates) and mitophagy (autophagy of damaged mitochondria). Specific cargo binding proteins that also interact with Lc3/GABARAP proteins (so-called autophagy receptors) have been identified and found to facilitate selective autophagy by connecting cargo to the autophagy membrane. Selective autophagy plays an important house-keeping function under basal nutrient-rich conditions to mediate the removal of superfluous or damaged organelles and protein aggregates that otherwise could be toxic. Zebrafish has been used to study degradation of mitochondria and protein aggregates in different neurodegenerative disorder models and to investigate the role of autophagy in protection against pathogens, as reviewed below. For most other types of selective autophagy zebrafish have either been not used at all or very scarcely used. 

### 7.1. Mitophagy

Selective removal of mitochondria is termed as mitophagy. The degradation of mitochondria by autophagy was already reported in the late 1950s when Clark and Novikoff observed mitochondria within membrane-bound compartments called “dense-bodies”, which were later shown to contain lysosomal enzymes [126,127]. The term mitophagy was coined by Lemasters and colleagues when they observed the engulfment of mitochondria into vesicular structures coated with Lc3 [128]. Mitophagy is also seen in yeast and this has helped dissect the molecular machinery required for the process [129,130]. Some of the proteins required for yeast mitophagy do not have a mammalian orthologue (e.g., Atg32, Atg11), but have functional homologues, e.g., the outer mitochondrial membrane protein NIX acts both like Atg32 and Atg11 [131]. Mitophagy has also been found to be important during key developmental processes, such as the maturation of reticulocytes, after which the matured red blood cells lack mitochondria [132,133].

The E3 ubiquitin ligase Parkin is a major player in mitophagy [134]. Mitochondrial recruitment of Parkin is mediated by the accumulation of PTEN-induced putative kinase protein 1 (PINK1) on depolarized mitochondria [135,136,137]. NIX has also been shown to promote Parkin translocation and thereby promote mitophagy in mouse embryonic fibroblasts [138]. Loss of function mutations in the gene encoding Parkin *(park2)* have been linked to Parkinson’s disease (PD) with loss of dopaminergic neurons in the substantia nigra, a region in the mid brain that is responsible for motor function [139]. Parkinsonian syndrome has also been shown in zebrafish morphants lacking *pink1* [140] and *park2* [141], with dopaminergic cell loss. A TILLING (targeting-induced local lesions in genomes) mutant for *pink1* also shows significant reduction in the number of tyrosine hydroxylase (TH)^+^ cells and a reduction in mitochondrial complex I activity [142]. Thus, mitophagy dysfunction or an inability to degrade damaged mitochondria leading to accumulation of mitochondrial damage is a likely cause of PD. 

Proteins involved in mammalian mitophagy are well conserved in zebrafish [143], which makes zebrafish a good model to further delineate the functional significance of mitophagy in vivo. There have not yet been many mitophagy studies in zebrafish, but several tools exist to study mitochondrial dynamics. One study tried to observe sites for mitophagy in Rohon Beard neurons of zebrafish where *UAS:LC3.GFP* was coinjected with *UAS:mitoTagRFP-T* into the *Isl2b:Gal4* transgenic line. Lc3 was found to colocalize with mitochondria, but proper mitophagy assays were not performed [144]. It would be highly interesting to see if Lc3 disappeared over time from these contact points. As mentioned earlier, a tandem-based approach to tagging mitochondrial proteins would help in observing their degradation kinetics via autophagy. It is a highly exciting time for zebrafish mitophagy studies. One major problem is however the lack of antibodies for zebrafish mitochondrial proteins, but with larger research interest churning up for mitochondrial studies, this scenario is likely to diminish fast.

### 7.2. Aggrephagy

Several neurodegenerative disorders and prion diseases are characterized by neuronal protein aggregates and inclusion body formation. Aggregates are formed due the accumulation of misfolded proteins [145]. Misfolded proteins can either be degraded by the ubiquitin–proteasome system (UPS), through chaperone-mediated autophagy (CMA) or by macroautophagy. Almost all soluble proteins (except for the long lived proteins) are turned over by the UPS, but as large protein-aggregates are difficult to degrade by the UPS, they are degraded by autophagy [146].

The zebrafish is a well-known model for the study of neurodegenerative disorders. Pharmacological modulation of autophagy in such zebrafish models of neurodegeneration has shown promising results. The first study to mention autophagy in zebrafish used a zebrafish Huntington’s disease (HD) model expressing EGFP-HDQ71 aggregates, where autophagy was found to be upregulated by reagents such as calpastatin, calpeptin, 2′5′DDA and clonidine (Table 2), resulting in a decrease in EGFP-HDQ71 aggregates [147]. HD is caused by glutamine expansions (polyQ) in the gene encoding the Huntingtin protein that make it prone to misfold and aggregate. In another study using the zebrafish HD model expressing EGFP-HDQ71 it was demonstrated that autophagy inducers like rapamycin and clonidine cleared the aggregate in the retina [148]. A zebrafish model of Alzheimer’s disease (AD) is characterized by neuronal tau aggregates and was found to have reduced aggregate clearance and decreased Lc3-II levels upon overexpression of phosphatidylinositol binding clathrin assembly protein (Picalm) [114]. PICALMs are known to interact with and thereby regulate the endocytosis of Soluble NSF Attachment Protein Receptor proteins (SNAREs), such as VAMP2, VAMP3 and VAMP8 [149]. In a recent study, it was seen that clonidine, rilmenidine and rapamycin had positive effects on the clearance of aggregated A152T-tau. It was also observed that transient overexpression of Atg5 upregulated autophagy in zebrafish larvae by 2 dpf, evident by an increase in lipidated Lc3-II and a reduction in hyperphosphorylated tau—one that causes aggregation of tau [108]. BAG3 is a key component of the chaperone-assisted selective autophagy (CASA) pathway [150]. It was recently found that in a transgenic zebrafish model of myofibrillar myopathy (induced by expression of a mutant of filamin C (FLNC^W2710X^-eGFP)) the BAG3-mediated CASA pathway is impaired and insufficient in clearing the FLNC^W2710X^ aggregates, and that autophagy promoting compounds like rapamycin or carbamazepine facilitated aggregate reduction [113].

The zebrafish as a model is proving to be essential for understanding disease mechanisms of several neurodegenerative disorders characterized by insoluble protein aggregates. There are a plethora of studies showing reduction of protein aggregates by the induction of autophagy. So far, molecular studies on the sequestration of aggregates into autophagosomes have not elucidated the role of different autophagy proteins during aggrephagy in zebrafish. This is very likely to change in the near future with the advent of CRISPR/Cas9 technology and the availability of antibodies for protein studies. The transparency of the zebrafish and its amenability to different drugs makes them an excellent model for neurodegenerative research.

### 7.3. Xenophagy

The role of autophagy as an anti-microbial mechanism was first demonstrated in studies by Yoshimori and co-workers, who showed that *Streptococcus pyogenes* multiplied in Atg5-deficient cells and by Deretic and co-workers, who showed that intracellular survival of *Mycobacterium tuberculosis* could be limited by starvation-induced or rapamycin-induced autophagy [151,152]. Since then autophagy has become recognized as a crucial defense mechanism against bacterial, viral, fungal, and protozoan pathogens [14]. Once internalized by host cells, microbial invaders often escape from phagosomes into the cytosol, where they become targets for xenophagy. Cytoplasmic microbes or damaged membranes of phagosomes are marked by molecular tags such as ubiquitin and galectins, which are the substrates for recognition by selective autophagy receptors that are also involved in mitophagy [153]. The autophagy receptors p62 and optineurin have been shown to protect against bacterial infections in zebrafish models [66,67]. Autophagy-related mechanisms distinct from xenophagy also play a role in host defense. In particular, Lc3 can be recruited directly to phagosomes in a process named Lc3-associated phagocytosis (LAP) [154]. Pathogens have evolved various strategies to evade xenophagy or LAP [155,156]. Zebrafish models for *Salmonella typhimurium* and *Mycobacterium marinum* infection have been used to study some of the virulence mechanisms that pathogens use to counteract the host autophagy response [119,120,157]. In addition, it has been shown that pharmacological stimulation of autophagy can improve the zebrafish host defense against *Mycobacterium marinum* infection [105,118].

**Figure 5:**
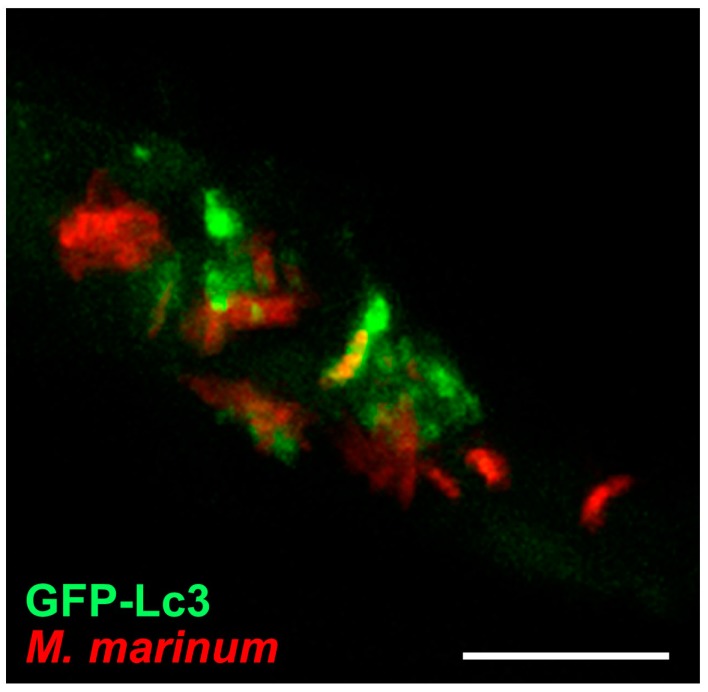
Confocal imaging of Tg(CMV:EGFP-Mapllc3b) on infection. GFP-Lc3 signal around clusters of *M. marinum* bacteria in 4-day-old zebrafish larva at 3 days post infection. Scale bars, 10 µM.

GFP-Lc3 transgenic zebrafish have been used to study bacterial infections with *Shigella flexneri* and *M. marinum* [67,77,102,158]. Both these pathogens have the ability to escape from phagosomes and replicate inside the cytosol. A proportion of cytosolic *Shigella* bacteria are trapped inside cage-like structures formed by septins, which are cytoskeletal components that prevent *Shigella* from actin tail formation and cell-to-cell spreading [159]. In vitro studies have shown that septin-caged *Shigella* are targeted to autophagy [159]. In agreement, in vivo imaging of zebrafish embryos demonstrated recruitment of GFP-Lc3 and the presence of bacteria in autophagosomes was confirmed by ultrastructural analysis [67]. Septin-caged *M. marinum* bacteria were also observed in zebrafish embryos, but the significance of septin caging in relation to autophagic targeting remains to be investigated [67]. Entrapment of *M. marinum* bacteria by GFP-Lc3 vesicles could be visualized by confocal time lapse imaging of infected zebrafish [92]. This study also revealed that GFP-Lc3 vesicles frequently appeared as puncta in close vicinity of single bacteria or bacterial clusters (Figure 5). Correlative light and electron microscopy confirmed that these vesicles represent autophagosomes, which might contribute to the delivery of ubiquitinated antimicrobial peptides to the *M. marinum* -containing compartments [92,160]. 

Dram1 is an autophagy modulator that is induced during infection of zebrafish by the MYD88-NFκB-dependent signaling pathway, which occurs downstream of pathogen recognition by Toll-like receptors [68]. Expression of zebrafish Dram1 can also be induced by the p53-stabilizing agent roscovitin, in agreement with the identification of human DRAM1 as a p53 target gene [68,161]. Overexpression of Dram1 by mRNA injection was found to result in increased lysosomal acidification of *M. marinum* containing compartments and to improve resistance of zebrafish embryos to the infection [68]. In addition, Dram1 overexpression enhances GFP-Lc3 recruitment to *M. marinum* and this function requires the cytosolic DNA sensor Sting and the ubiquitin receptor p62. In agreement, morpholino knockdown of Dram1 reduced GFP-Lc3 recruitment to *M. marinum* and impaired host defense [68]. Dram1 is a member of a conserved family of transmembrane proteins and localizes predominantly to lysosomes [161]. Its precise mechanism of action is currently unknown, but a recent study on a human family member, DRAM2, suggests an interaction with the Beclin1-Vps34-UVRAG complex, which leads to displacement of the inhibitor Rubicon and thereby enhanced PI3K activity [162]. Since *M. marinum* infection in zebrafish mimics aspects of human tuberculosis, further research into the Dram1-mediated selective autophagy pathway could help to develop novel strategies for host-directed anti-tuberculosis therapy [160].

Recently, the zebrafish has also been used to study the host autophagic response to a viral infection [109]. Zebrafish can be infected with spring viremia of carp virus (SVCV), a member of the rhabdovirus family. Infection with this virus induces the production of Tnfα, a potent pro-inflammatory cytokine that normally serves a host-protective function, but is exploited by certain viruses to their benefit. GFP-Lc3 imaging and Western blot analysis showed that depletion of Tnfα increased autophagy in SVCV-infected larvae. Since depletion of Tnfα also improved resistance to SVCV infection, the authors concluded that inhibition of autophagy is the mechanism behind the deleterious effect of Tnfα on viral clearance [109]. A wide variety of other zebrafish infection models provide excellent tools to further advance our understanding of the role of autophagy in host-pathogen interactions [163].

## 8. Future Perspective

The zebrafish is fast becoming one of the best vertebrate models for studying disease states and conditions. Owing to the various advantages that they pose and the ease at which the present advancements in genome editing technology can be applied, zebrafish hold unparalleled potential for all basic and translational research. Existing studies of autophagy in zebrafish have presented invaluable insight into the role of autophagy in development, disease progression and drug discovery. There is still however a need for antibodies that recognize specific zebrafish autophagy proteins, and their modifications (at present a limitation). The contribution of CRISPR/Cas9 to scientific research has been immense, but the overall technology depends upon efficient sgRNAs and thus having a database system to maintain and expand the existing sgRNAs is a must. Autophagy research has been expanding and the vitality of autophagy as a degradation system has been acknowledged worldwide with Yoshinori Ohsumi receiving the Nobel Prize in Medicine or Physiology in 2016. Autophagy research using the zebrafish as a model system looks promising for many more breakthroughs and new therapeutics against many diseases. 

## Figures and Tables

**Table 1:** Zebrafish (*Danio rerio*) orthologues of human autophagy genes, with amino acid percentage identity and allele availability at the Sanger ZMP. ULK: UNC-51-like kinase; ZMP: Zebrafish Mutation Project; PE: phosphatidylethanolamine, mTORC1: Mammalian Target of Rapamycin Complex 1, AMPK: Adenosine Mono-Phosphate Kinase; ER: Endoplasmic Reticulum; PtdIns3K: Phosphatidylinositol 3-Kinase; VPS34: Vacuolar Protein Sorting 34.

Core Autophagic Process		Mammalian Protein	Zebrafish Orthologue	Refseq Id of Zebrafish DNA/Protein	Ensemble Id of Zebrafish DNA/Protein	Amino Acid Identity	Role in Autophagy	Mutant Allele Availability at the Sanger ZMP
**Nucleation step**	ULK1 complex	ULK1	*ulk1a*	NM_001130631, NP_001124103.1	ENSDART00000090534.4	50%	Phosphorylated by mTORC1 (negative) and AMPK (positive). Induces autophagy by phosphorylation of ATG13	Ulk1a—Yes
			*ulk1b*	XM_005161121.3, XP_005161178.1	ENSDART00000112407.3			No
		ULK2	*ulk2*	XM_002664615.4, XP_002664661.3	ENSDART00000153726	74%		No
		ATG13/KIAA0652	*atg13*	NM_200433, NP_956727	ENSDART00000052324.5	71%	Member of the ULK1 complex, phosphorylated by mTORC1 and ULK1	No
		Fip200/RB1CC1	*rb1cc1*	XM_009302198.2, XP_009300473.1	ENSDART00000113014.3	59%	Scaffold for ULK1/2 and ATG13	Yes
		ATG101	*atg101*	NM_001037239, NP_001032316	ENSDART00000063544.6	87%	Interacts with ATG31	No
	Class III PI3-kinase complex (PIK3C3)	ATG14L	*atg14L/kiaa0831*	NM_001024812, NP_001019983	ENSDART00000018683.10	67%	Autophagy-specific subunit of PIK3C3 complex I. ER binding motif	Yes
		PtdIns3K/VPS34	*pik3c3*	NM_001328533, NP_001315462	ENSDART00000101265.4	87%	Catalytic subunit. Phosphorylates phosphatidylinositol to generated PI3-phosphate	No
		Beclin1	*beclin1*	NM_200872, NP_957166	ENSDART00000115237.3	79%	Subunit of PIK3C3. Regulatory function through binding to Bcl-2	Yes
		p150	*pik3r4*	XM_005158299.3, XP_001922676.1	ENSDART00000085228.5	82%	Adaptor protein for VPS34	No
**Atg12 conjugation system**		ATG12	*atg12*	NM_001246200, NP_001233129	ENSDART00000101304.4	71%	Ubiquitin like, conjugates to ATG5	Yes
		ATG7	*atg7*	XM_017358254.1, XP_017213743.1	ENSDART00000162152	77%	E1-like enzyme	Yes
		ATG10	*atg10*	NM_001037124, NP_001032201.1	ENSDART00000160159.1	50%	E2-like enzyme	No
		ATG5	*atg5/apg5L*	NM_205618, NP_991181	ENSDART00000029727.6	81%	Conjugated by ATG12	Yes
		ATG16L1	*atg16L1*	NM_001017854, NP_001017854	ENSDART00000161937.1	69%	Interacts with ATG5 to form the ATG12-5-16L1 complex, an E3 like ligase for Atg8 conjugation	No
**Atg8 conjugation system**		MAP1-Lc3A	*map1-lc3a*	NM_214739, NP_999904	ENSDART00000042322.3	96%	Ubiquitin like, conjugates to PE	No
		MAP1-Lc3B	*map1-lc3b*	NM_199604, NP_955898	ENSDART00000163508.1	93%		
		MAP1-Lc3C	*map1-lc3c*	NM_200298, NP_956592	ENSDART00000161846.2	72%		
		GABARAP	*gabarapa*	NM_001013260, NP_001013278	ENSDART00000051547.3	98%	Ubiquitin like, conjugates to PE	No
		GABARAPL1	*gabarapl1*	NM_001002707, NP_001002707	ENSDART00000060037.3	59%		
		GABARAPL2	*gabarapl2*	NM_205723, NP_991286	ENSDART00000039485.6	97%		
		ATG4A	*atg4a*	NM_001024434, NP_001019605	ENSDART00000026666.10	70%	Atg8 C-terminal hydrolase, deconjugating enzyme	Yes
		ATG4B	*atg4b*	NM_001089352, NP_001082821	ENSDART00000121558.3	73%		No
		ATG4C	*atg4c*	NM_001002103, NP_001002103	ENSDART00000051779.3	59%		Yes
		ATG4D	*atg4da*	XM_009294436.2, XP_009292711.1	ENSDART00000152289.2	50%		No
			*atg4db*		ENSDART00000172196	50%		No
		ATG3	*atg3*	NM_200022, NP_956316	ENSDART00000041304.7	82%	E2-like enzyme	No
**Other core Atg proteins during autophagosome formation**		ATG2A	*atg2a*	XM_009307758.2, XP_009306033.1	ENSDART00000172444.1	55%	Proper closure of autophagosome	No
		ATG2B	*atg2b*	XP_001340508.3	ENSDART00000155615	42%		No
		ATG9A	*atg9a*	NM_001083031, NP_001076500	ENSDART00000065411.6	71%	Transmembrane protein on the autophagsome	No
		ATG9B	*atg9b*	NM_001320078, NP_001307007	ENSDART00000147499.3	49%		No
		WIPI1	*wipi1*	NM_200391, NP_956685	ENSDART00000059533.4	71%	Phosphatidyl-insolitol 3-phosphate PI(3)P-binding proteins	Yes
		WIPI2	*wipi2*	NM_001327789, NP_001314718	ENSDART00000134026.2	82%		Yes
		WIPI3/WDR45B	*wipi3/wdr45b*	NM_200240, NP_956534	ENSDART00000152327.2	96%		No
		WDR45	*wipi4*	NM_200231, NP_956525	ENSDART00000130229.2	90%		No
**Autophagy receptor proteins**		NCOA4	*ncoa4*	NM_201129, NP_957423	ENSDART00000017052.8	38%	Autophagy cargo receptor required during iron homeostasis	No
		SQSTM1/p62	*sqstm1/p62*	NM_001312913, NP_001299842	ENSDART00000140061.2	44%	Autophagy cargo receptor	No
		OPTN	*optn*	NM_001100066, NP_001093536	ENSDART00000014036.10	41%	Autophagy cargo receptor	No
		CALCOCO2/NDP52	*calcoco2*	NM_001020741, NP_001018577	ENSDART00000152964.2	30%	Autophagy cargo receptor during xenophagy and mitophagy	No
		NBR1	*nbr1*	NM_001305595, NP_001292524	ENSDART00000133048.2	38%	Autophagy cargo receptor	Yes
		TAX1BP1	*tax1bp1a*	NM_001346178, NP_001333107	ENSDART00000171664.1	44%	Autophagy cargo receptor during mitophagy	Yes
			*tax1bp1b*	NM_212664, NP_997829	ENSDART00000040727.7	52%	Autophagy cargo receptor	Yes

**Table 2:** Constitutive and transient reporter constructs used to study autophagy in zebrafish.

Reporter	Expression	Reference
Tg(CMV:GFP-Lc3)	Ubiquitous	[86]
Tg(CMV:GFP-Gabarap)	Ubiquitous	[86]
Tg(pT2-mCherry-Sqstm1)	Ubiquitous	[45]
Tg(pT2-Lamp1-mCherry)	Ubiquitous	[45]
Tg(TαCP:mCherry-GFP-Map1lc3b)	Cone photoreceptors	[97]
Tg(TαCP:GFP-Map1lc3b)	Cone photoreceptors	[97]
Tg(TαCP:YFP-2XFYVE)	Cone photoreceptors	[97]
Tg(CMV:EGFP-Map1lc3b; CMV:mCherry-Map1lc3b)	Ubiquitous	[46]
Tg(CMV:EGFP-Gabarapa; CMV:mCherry-Map1lc3b)	Ubiquitous	[46]
Tg(fabp10: EGFP-Map1lc3b)	Liver	[95]
Tg(TαCP:GFP-Map1lc3b)	Cone photoreceptors	[90]
pEGFP–Map1lc3b	Transient (embryonic cells)	[103]
mCherry-Lc3 mRNA	Transient	[104,105]
pDest(CMV:RFP.GFP.Lc3) mRNA	Transient	[105]
GFP-Lc3-RFP-Lc3ΔG mRNA	Transient	[101]
mCherry-Map1lc3b	Transient	[106]
hsp70l:RFP-Map1lc3b	Transient	[61]

**Table 3:** List of antibodies ever used to detect autophagy-related proteins in zebrafish. (Catalogue numbers listed in italics have been used for immunostaining too).

Antibody	Company	Catalogue No.	Reference
L_C_3	Novus biologicals	NB100-2220	[93,108,111,112,113,114,115,116]
	Novus biologicals	NB100-2331	[86,94,117]
	Proteintech	12135-1-AP	[118]
	Cell Signaling	4108	[45,109]
		Not indicated	[74,104]
		2775	[62,114]
	MBL	Not indicated	[119]
		PD014	[95]
		PM036	[115]
	Sigma	L7543	[59]
	Abcam	*ab51520*	[106]
	Thermo Scientific	*PA1-46286*	[68]
Gabarap	Non-commercial		[86]
SQSTM1/p62	Abnova	H00008878-M01	[111]
	Cell Signaling	5114	[94,112]
	Abcam	ab109012	[117]
		*ab31545*	[68]
	MBL Japan	Not indicated	[119,120]
	Cliniscience	PM045	[67]
mTOR	Cell Signalling	2983	[116]
Phospho-mTOR, Ser2448	Cell Signaling	2971	[121]
Akt	Cell Signaling	Not indicated	[74]
Phospho-Akt, Ser473	Cell Signaling	9271	[74,121]
Phospho-S6K, Thr389	Cell Signaling	9205	[121]
Phospho-S6K	Cell Signaling	Not indicated	[104]
S6k	Cell Signaling	2708	[121]
Beclin1	R&D systems	Not indicated	[120]
	Abcam	Not indicated	[104]
	Santa Cruz	H-300 11427	[58,62]
Lamp-2A	Abcam	ab18528	[121]
Atg5	Novus biologicals	NB110-53818	[59,93]
	Abcam	Not indicated	[108]
		ab540333	[59]
	Abgent	AP1812a, AP1812b	[59]
Actin (loading control)	Sigma	Not indicated	[108]
α-Tubulin (loading control)	Sigma	T5168	[73]
GAPDH (loading control)	Millipore	Not indicated	[108]

**Table 4:** List of reagents used to modulate autophagic activity in zebrafish (post–2014).

Reagent	Conc.	Observed Effect	Reference
**Reagents increasing autophagy**			
Rapamycin	400 nM	Inhibited mTOR, activated autophagy; ameliorated kidney cysts and preserved kidney function	[112]
	1 µM	Increased autophagy dependent release of Tumor necrosis factor α and Interleukin-8 (TNFα and IL-8) in mycobacterium-infected zebrafish larvae	[105]
	10 µM	Enhanced clearance of protein aggregates in FLNC^W2710X^ mutants	[113]
	30 µM	Enhanced the clearance of A152T-tau, reduced hyperphosphorylated tau	[108]
Torin1	0.4 µM	ATP-competitive mTOR inhibitor; increased Lc3-I and Lc3-II levels; increased resistance of zebrafish embryos to *Salmonella* Typhimurium infection	[119]
Rilmenidine	50 µM	Imidazoline-1 receptor agonist, reduced cyclic adenosine monophosphate (cAMP) levels; enhanced the clearance of A152T-tau	[108]
Clonidine	30 µM	Imidazoline-1 receptor agonist, reduced cAMP levels; enhanced the clearance of A152T-tau	[108]
Carbamazepine	20 µM	mTOR-independent autophagy activator; attenuated kidney cysts	[112]
	50 µM	Increased autophagy-dependent cytokine release	[105]
	0.5 mM	Enhanced clearance of protein aggregates in FLNC^W271^°^X^ mutants	[113]
Minoxidil	400 nM	Inhibited L-type Ca^2+^ channel currents, thereby activating autophagy via a cyclical mTOR independent pathway; attenuated kidney cysts	[112]
Auten-67	50 µM	Upregulated autophagy by inhibiting phosphatase activity of MTMR14, which is a negative regulator of autophagic membrane formation.	[125]
Spermidine	5 mM	Inhibited acetyl-transferases; enhanced clearance of protein aggregates in FLNC^W2710X^ mutants	[113]
Trifluoperazine (TFP)	1 mM	Activated Transcription Factor EB (TFEB) which is a master regulator of autophagy pathway, activated autophagy	[111]
**Reagents blocking autophagosome—lysosome fusion**			
Bafilomycin A1 (BafA1)	20 nM	Autophagosome-lysosome fusion inhibitor; slight increase in Lc3-II	[117]
	25 nM	Significant increase in Lc3-II	[104]
	167 nM	Showed defects in autophagy flux	[112]
	200 nM	Zebrafish larvae recapitulated atp6v0ca morphant, reduced yolk opacity and senescence phenotypes	[45]
Chloroquine	10 µM	Autophagosome-lysosome fusion inhibitor; blocked autophagy and increased GFP-Lc3 punctae	[73]
	2 mM	Reduced muscle regeneration on blocking autophagy	[94]
	100 c	Decreased Lc3 accumulation, defective autophagy	[113]
	5 µM	Increased Lc3 accumulation in Kri1l*^cas002^* mutant	[104]
	2.5 µM	Significant accumulation of autophagosomes in zebrafish larvae infected with mycobacterium	[105]
	50 µM	Accumulation of Lc3-II and p62; no effect on zebrafish infection with *Salmonella* Typhimurium	[119]
Omeprazole	100 µM	Late-stage autophagy inhibitor; rescued senescence phenotype	[45]
Lansoprazole	100 µM	Late-stage autophagy inhibitor; rescued senescence phenotype	[45]
Pantoprazole	100 µM	Late-stage autophagy inhibitor; rescued senescence phenotype	[45]
Pepstatin A	5 µg/mL	Prevented autolysosomal maturation and turnover	[45]
E-64d	5 µg/mL	Prevented autolysosomal maturation and turnover	[45]
Ammonium chloride	100 mM	Prevented autolysosome maturation; blocked autophagy and increased GFP-Lc3 punctae	[113]
	100 mM	Significant increase in Lc3-II	[117]
**Early autophagy inhibitor**			
3-MA	10 mM	Inhibited PIK3C3 activity; significant reduction of autophagy visualized by Lc3-II puncta	[104]
